# The Future of the Army Medical Service

**Published:** 1900-08-25

**Authors:** 


### THE FUTURE OF THE ARMY MEDICAL SERVICE.

Mr. Wyndham lately made a statement in the House of Commons to the effect that, instead of trying to expand the Army Medical Corps in time of peace, so that it would be adequate in time of war, it would be necessary to have a highly efficient army corps as a directing body, and in time of peace to make application to the great medical bodies in this country to know what men would be ready in time of war to come forward and work for the Army Medical Corps. Criticising this view of the matter, the *British Medical Journal* points out that, while the Army Medical Service is, of course, needed as a directing body in time of war, and will, no doubt, always have to undertake the direction of such civil help as may then be required, the great fact remains that even in time of peace the service has of late years been much undermanned. The demand for an increase in the personnel of the service is not founded upon any wish to maintain in times of peace a medical service large enough to cope with a considerable war, but on the fact that for long back the service has been starved. The great cause of the continued unpopularity of the Army Medical Service in the medical schools is the unfair and harassing high-pressure conditions under which it is constantly worked; these are felt in inequitable tours of foreign service, perpetual movements from station to station, difficulty in obtaining private or study leave, grinding orderly duty, &c. The first thing wanted is to strengthen the corps by, say, 200 medical officers, so that it may be reasonably equal to carry on peace duties and minor wars.

